# The ghost of hosts past: impacts of host extinction on parasite specificity

**DOI:** 10.1098/rstb.2020.0351

**Published:** 2021-11-08

**Authors:** Maxwell J. Farrell, Andrew W. Park, Clayton E. Cressler, Tad Dallas, Shan Huang, Nicole Mideo, Ignacio Morales-Castilla, T. Jonathan Davies, Patrick Stephens

**Affiliations:** ^1^ Department of Ecology and Evolutionary Biology, University of Toronto, Toronto, ON, Canada; ^2^ Odum School of Ecology, University of Georgia, GA, USA; ^3^ School of Biological Sciences, University of Nebraska, Lincoln, NE 68588, USA; ^4^ Department of Biological Sciences, Louisiana State University, Baton Rouge, LA 70806, USA; ^5^ Department of Biological Sciences, University of South Carolina, Columbia, SC, 29208, USA; ^6^ Senckenberg Biodiversity and Climate Research Centre (SBiK-F), Frankfurt am Main, Germany; ^7^ Universidad de Alcalá, GloCEE - Global Change Ecology and Evolution Research Group, Departamento de Ciencias de la Vida, 28805 Alcalá de Henares, Madrid, Spain; ^8^ Department of Botany, University of British Columbia, Vancouver, BC, Canada, V6T 1Z4; ^9^ Department of Forest and Conservation Sciences, University of British Columbia, Vancouver, BC, Canada, V6T 1Z4; ^10^ Department of Botany and Plant Biotechnology, African Centre for DNA Barcoding, University of Johannesburg, Johannesburg 2092, South Africa

**Keywords:** host–parasite interaction, phylogenetic ecology, coextinction, virulence evolution, infectious diseases

## Abstract

A growing body of research is focused on the extinction of parasite species in response to host endangerment and declines. Beyond the loss of parasite species richness, host extinction can impact apparent parasite host specificity, as measured by host richness or the phylogenetic distances among hosts. Such impacts on the distribution of parasites across the host phylogeny can have knock-on effects that may reshape the adaptation of both hosts and parasites, ultimately shifting the evolutionary landscape underlying the potential for emergence and the evolution of virulence across hosts. Here, we examine how the reshaping of host phylogenies through extinction may impact the host specificity of parasites, and offer examples from historical extinctions, present-day endangerment, and future projections of biodiversity loss. We suggest that an improved understanding of the impact of host extinction on contemporary host–parasite interactions may shed light on core aspects of disease ecology, including comparative studies of host specificity, virulence evolution in multi-host parasite systems, and future trajectories for host and parasite biodiversity.

This article is part of the theme issue ‘Infectious disease macroecology: parasite diversity and dynamics across the globe’.

## 1. Introduction

The Earth's biodiversity is in the midst of a crisis, with current rates of extinction that are conservatively 100 times faster than the normal background rate [1]. Yet we are only beginning to understand the true scope of this crisis. Mammals are among the most well-documented groups, and over a quarter of all mammal species are threatened with extinction [2]. The loss of any one species will also impact affiliated species, including mutualists, commensals and parasites, and when associations are obligate, we risk cascading extinctions. The intimate interactions between parasites and their hosts have led to the suggestion that parasites may comprise the majority of endangered species [3], and increasing advocacy for the inclusion of parasites in global conservation planning [4]. Yet even within mammals, one of the best-sampled host groups, it is unclear how many parasite species may be lost with future host extinctions [5,6], what effect losses of hosts and their parasites will have on the ecological structure of communities or how patterns that we observe in contemporary communities may be related to losses of hosts and parasites in the recent past.

Parasites play critical roles in ecological communities through impacts on host populations and structuring food webs [7,8]. However, in comparison with their hosts, parasite extinctions are notoriously difficult to observe [9], though some can be inferred through analysis of ancient samples [10,11], or through co-phylogenetic analyses [12]. Beyond direct observation, the coextinction of parasites along with the loss of their hosts [13] has been studied via comparative analyses of threatened and non-threatened hosts [14,15], and simulations that identify likely coextinctions resulting from future host extinctions [3,16–19]. Both of these approaches commonly assume a complete extinction of parasites when, and only when, all of their documented hosts have gone extinct [20]. However, multi-host parasites may require multiple hosts to maintain a net reproductive rate greater than one, suggesting that the extinction of even a single host may imperil a parasite [21–23]. Moreover, the extinction of some of their hosts also impacts the ecology and evolution of multi-host parasites through altering the adaptive landscape across available hosts [24]. Host extinction therefore not only has the potential to result in parasite extinction, but may alter host specificity and shift the evolutionary landscapes shaping future parasite evolution. Predicting the impacts of host extinctions on host specificity becomes especially muddied when we expand our notion of host specificity beyond the number of host species infected.

Host specificity is a fundamental property of parasites and can be quantified by the richness, evenness or the ecological or evolutionary diversity of host species that a parasite infects [25]. Parasite species can display various degrees of specificity, from infecting a single host species (i.e. a specialist parasite) to infecting multiple host species (i.e. a generalist parasite). Among generalist parasites, the degree of specificity can also vary dramatically. Using phylogenetic distances among hosts to measure specificity, a parasite infecting the same number of hosts may infect only closely related hosts or infect hosts from across multiple, distantly related clades [25,26]. The degree of host specificity is a product of historical associations of parasites with their hosts, including processes of co-speciation and parasites shifting to infect novel hosts [27,28]. Identifying the set of host species that a parasite could infect given suitable opportunity (i.e. the potential host range of a parasite) allows us to infer ancestral host–parasite associations [29] and make crucial predictions of the potential for emergence in novel hosts [30,31] and likely impacts following cross-species transmission [32–34].

Predictions of unobserved host–parasite associations are often based on an assumption that present-day associations accurately reflect potential host ranges [31,35,36]. However, host range is a dynamic property of parasites that evolves through cospeciation, host shifts, and the gains and losses of hosts over varying timescales [37–41]. Changes in parasite host specificity as a result of host-switching and shifting geographic ranges have attracted considerable attention by researchers [42–47], whereas extinction history has tended to be overlooked. Similar to the impact of host-switches, if recent historical host extinctions have reshaped contemporary host–parasite associations, we may be misled as to the intrinsic specificity of parasites. For example, the extinction of an evolutionarily distinct host may shift our perception of a parasite from being a phylogenetic generalist to a phylogenetic specialist. We use the term ‘apparent specificity’ to reflect host specificity inferred from current documented host–parasite associations. Identifying the ways in which host specificity may have been influenced by past host extinction is important for quantifying risks of parasites establishing on novel hosts, and predicting how selection on multi-host parasites may shift in response to future host extinctions.

Here, we examine how host extinction may shape patterns and perceptions of host specificity and alter emergent patterns of parasite diversity and distribution at broader scales. We first summarize theoretical predictions on the consequences of host extinction, then showcase examples of these through the lenses of both historical mammal extinctions and projected future extinctions based on contemporary threat status. Although these patterns are complex, we highlight how host extinction can lead to both increases and decreases in apparent parasite host specificity, demonstrate how host specificity may be impacted by non-random host extinction and consider implications for projecting how host specificity might respond to future host extinctions. Finally, we discuss the impacts of host extinction on parasite ecology and evolution, with a focus on altering costs of generalism versus specialism, parasite fitness, transmission potential and virulence evolution. While current coextinction theory largely addresses parasite extinction resulting from host extinction, we suggest that expanding this framework to include contemporary measures of host specificity and theory underlying co-adaptation and virulence evolution in multi-host systems will be crucial to understanding how biodiversity loss impacts infectious diseases more broadly.

## 2. Proximate impacts of host extinction on parasite host specificity

**Figure 1.**
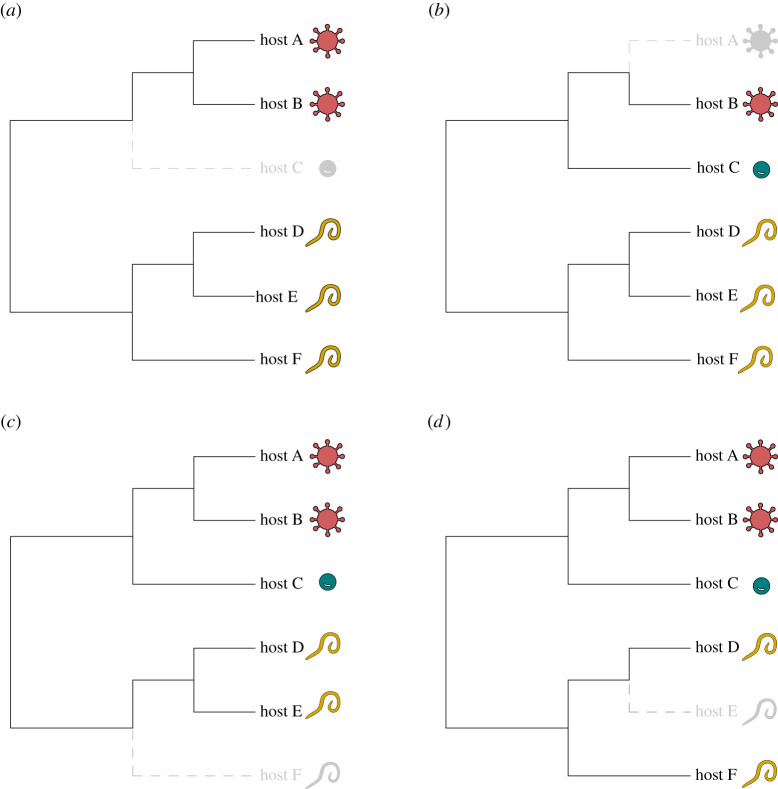
Examples of how host extinction can impact parasite specificity. Each shape represents a hypothetical parasite species, with their positions reflecting interactions with hosts alongside the host phylogeny. Each extinction scenario involves the loss of one host species (depicted by grey dashed lines). Depending on the original set of host–parasite interactions, the extinction of a host species may result in the loss of a single-host parasite, an example of coextinction (*a*), the reclassification of a former generalist to a single-host parasite (*b*), or more subtle changes in which the average phylogenetic distances among hosts may decrease (*c*) or increase (*d*) among the remaining hosts. (Online version in colour.)

The concept of parasite coextinction was first formulated as the extinction of a host-specific parasite with the loss of its sole host [13,48] (figure 1*a*). While assumed to be quite common, coextinction events are rarely documented [49]. A classic example of coextinction is the loss of the host-specific California condor louse (*Colpocephalum californici*) which went extinct after California condors (*Gymnogyps californianus*) became extinct in the wild and surviving individuals were deloused during a captive breeding and reintroduction programme [50]. Beyond coextinction, host extinction may result in a formerly multi-host parasite being constrained to infect a single host species (figure 1*b*). This was the case for two species of passenger pigeon louse (*Columbicola extinctus* and *Campanulotes defectus*) that parasitized both the passenger pigeon (*Ectopistes migratorius*) and another closely related species [3,51]. Ironically, this was initially presented as a classic example of parasite coextinction as these two parasite species had only been described on the passenger pigeon and were presumed extinct with the pigeon [13], and only later were they found alive and parasitizing another host species. In hindsight, if the full host ranges had been known, these parasites would have been considered to be multi-host parasites and now constrained to single-host specialists after the extinction of the passenger pigeon. For parasites that infect more than two hosts, host extinction in the absence of host jumps will always reduce host richness, thus increasing perceived taxonomic specialization. However, the loss of a host species may increase or decrease the average phylogenetic distances among extant hosts (figure 1*c,d*), shifting our perception of the phylogenetic host breadth of the parasite. The directionality of the shift in phylogenetic host breadth is highly context dependent, which we explore further below.

## 3. Ghosts of hosts past

Building a greater understanding of coextinction and our perceptions of contemporary patterns of the host specificity of parasites may be achieved through studies of historical host extinctions. Looking to the past, we may find support for parasite extinctions following known host extinctions and identify cases in which historical extinctions likely influenced contemporary host specificity. As host species are pruned from the tree of life, those that survive can become increasingly isolated in the phylogeny, especially if they are nested within clades where extinction has been rampant [52]. The apparent phylogenetic specificity of the parasites found on them will therefore also change over time.

One approach to quantify how host extinction drives the phylogenetic distances among species is through the measure of evolutionary distinctiveness (ED) [53]. This measure, widely used in conservation prioritization [53–57], divides the total branch lengths of a phylogenetic tree among the tips. Each species is apportioned an amount of phylogenetic diversity, typically measured in millions of years of evolution, based on the sum of the branch lengths from the tip to the root of the tree, discounted by the number of shared descendents subtending from each branch. In this way, species that branched off deeper in the tree and have few or no extant relatives are considered to have high ED, whereas species in a young clade that recently underwent rapid speciation without much extinction would have low ED.

ED has been shown to be negatively related to parasite species richness per host [26,58], indicating that hosts more isolated in the mammal phylogeny have fewer parasites. This may result from different (and non-mutually exclusive) processes. The phylogenetic distance among hosts is negatively related to the propensity for parasite sharing [59–61], such that hosts isolated in the phylogeny may be less likely to be infected by multi-host parasites. One mechanism for this is the tendency for high ED hosts to have unique physiologies or life histories which may make them less likely to gain parasite species via host-switching events [62]. A less considered explanation is that more evolutionarily distinct hosts may have lost parasites because of the extinction of closely related species which acted as maintenance hosts. Following from the idea that single-host parasites will be lost with the extinction of their sole hosts, clades that have undergone large numbers of species extinctions are likely to have seen the coextinction of multi-host but clade-specific parasites. Thus, surviving hosts have both fewer close relatives (high ED) and fewer clade-specific parasites which would otherwise be maintained in more species-rich clades via frequent cross-species transmission.

While increasing ED may result in a reduction in parasite species richness per host, the remaining parasites may become apparent phylogenetic specialists or generalists depending on the initial host–parasite interactions before extinction (figure 1). For example, the loss of a host's close relatives might leave parasites stranded on these newly isolated hosts, if they are unable to evolve to infect additional host species (see [63]). In this case, host extinction may result in an increase of single-host parasites on distinct hosts (figure 1*b*) or they may appear to have lowered phylogenetic host specificity if parasite populations still persist on more distantly related hosts (figure 1*d*). Alternatively, if evolutionarily distinct hosts are more likely to be threatened with extinction [64], these hosts today may have already undergone severe population declines in the recent past, and thus host fewer specialist or generalist parasites, depending on host and parasite life histories [14,15].

**Figure 2.**
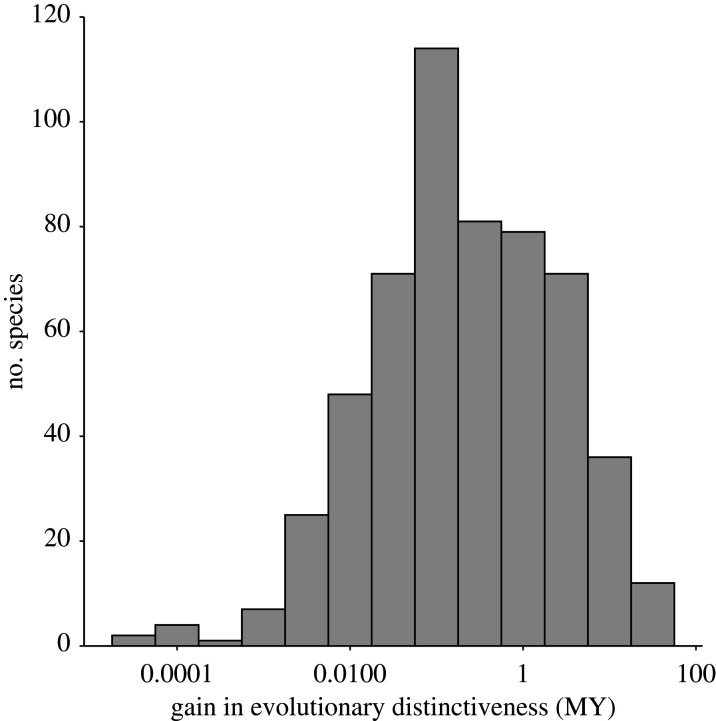
Distribution of gains in ED for extant mammal species resulting from mammal extinctions over the past 130 000 years. Gains in ED were calculated using the ‘equal-splits’ approach [53,72] and by subtracting contemporary ED measures per species from ED calculated including extinct taxa. Data from the PHYLACINE dataset [71] and Faurby & Svenning [70]

To explore empirical examples in which host extinction may have impacted contemporary patterns of host specificity, we pair a global database of contemporary mammal host–parasite interactions ([65], based on data amalgamated from [66–69]) with data on mammal host extinctions [70] and the Phylogenetic Atlas of Mammal Macroecology (PHYLACINE) [71]. PHYLACINE includes harmonized data on mammal traits, geographic distributions and phylogenetic relationships for all mammals since the last interglacial period (approx. 130 000 years ago until present), including extinct species. We use these data to identify illustrative examples, and demonstrate concepts that may be expanded upon to investigate the impact of host extinction on parasite specificity. With these data, we can calculate the ED of species before and after extinction, taking their difference as a measure of gains in ED and their increasing phylogenetic isolation. Over this time period, there are 352 documented mammal extinctions, which resulted in ED shifts for 551 extant mammals (figure 2). The majority of these ED gains are less than 1 million years (figure 2), but some species have seen large gains in ED on the order of tens of millions of years of added distinctiveness (table 1). As these hosts have lost close relatives, we suggest that the impacts of historical host extinction on parasite host specificity may be gleaned from investigating the ecology and evolution of parasites surviving on them. In the next section, we use a case study of an elephant tapeworm to demonstrate how this approach may generate new hypotheses of how host extinction may impact host specificity through altering parasite distributions, and ultimately shift selection pressures on surviving parasites.

**Table 1.** Extant mammal species with the largest gains in ED over the past 130 000 years (see figure 2 for the full distribution).

species	common name	ED extant	ED pre-extinction	ED gain
*Elephas maximus*	Asian elephant	47.69	10.00	37.69
*Solenodon cubanus*	Cuban solenodon/almiqui	66.45	32.60	33.85
*Dugong dugon*	dugong	60.50	30.86	29.64
*Loxodonta africana*	African bush elephant	47.69	19.86	27.83
*Macrotis lagotis*	greater bilby	45.85	19.73	26.12
*Tachyglossus aculeatus*	short-beaked echidna	74.61	49.04	25.57
*Hippopotamus amphibius*	common hippopotamus	33.28	9.14	24.14
*Zaglossus bruijnii*	western long-beaked echidna	39.05	16.62	22.43
*Tapirus indicus*	Malayan tapir	40.52	20.37	20.15
*Choloepus didactylus*	Linnaeus's two-toed sloth	25.59	7.31	18.27

## 4. Geographic discontinuity and the mystery of the elephant tapeworm

The species with the largest increase in ED is the Asian elephant (*Elephas maximus*), the only extant member of its genus. The Asian elephant is more closely related to extinct mammoths than African elephants (*Loxodonta africana*) [73], another species with large ED gains over the past 130 000 years (table 1). Currently listed by the IUCN as Endangered and with a declining population trend [74], Asian elephants are known to host at least 36 parasite species, 22 of which are only documented with this host species [65]. Among these parasites is the elephant tapeworm (*Anoplocephala manubriata*). Both Asian and African elephants are host to the eponymous cestode [75,76], even though these host species live on different continents, with no part of their geographic ranges overlapping. This raises a number of questions as to the ecology and evolutionary history of *A. manubriata*, and how disconnected species across the globe are infected by the same parasite. Although the taxonomy and biology of this parasite are rarely studied, the elephant tapeworm has been shown to use oribatid mites as obligate intermediate hosts [75], and phylogenetic analysis of tapeworms taken from Asian elephants were placed as sister taxa to *Anoplocephala* sp. infecting equids [76].

One possible explanation for the unusual distribution of *A. manubriata* might be circumglobal transmission. Some intermediate hosts of elephant tapeworms have distributions that span continents [75]. As oribatid mites commonly occur in soil communities, their general mechanisms of dispersal are relatively unknown, but some species have the ability to survive long-distance wind dispersal [77] and are speculated to undergo trans-oceanic dispersal via seabirds or ocean currents [78]. Although tapeworm populations in Asian and African elephants may be connected through rare cross-continental dispersal events, an alternative (and non-mutually exclusive) explanation is that the host range of the elephant tapeworm we see today is a relic of historical host extinctions.

**Figure 3.**
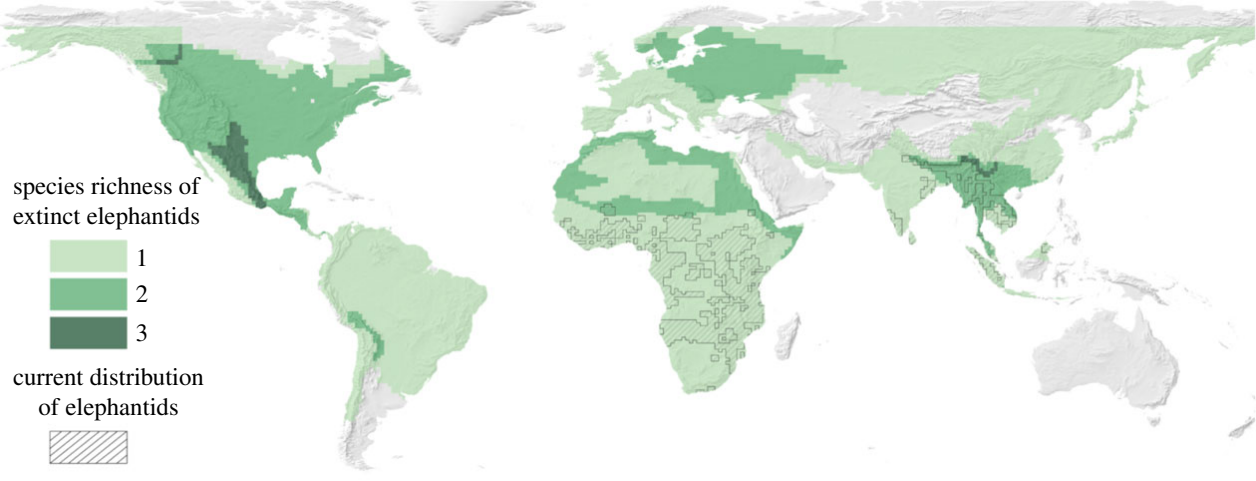
Distributions of species richness of extinct species from the Elephantidae family (green shades) and of current species of elephants (black stripes). The distribution of Asian elephants and African elephants would not be bridged by extinct elephantids in today's climate. Extinct species include *Cuvieronius hyodon, Elephas antiquus, Elephas cypriotes, Elephas iolensis, Elephas maximus, Elephas mnaidriensis, Elephas namadicus, Elephas naumanii, Elephas tiliensis, Loxodonta africana, Mammut americanum, Mammuthus columbi, Mammuthus exilis, Mammuthus primigenius, Notiomastodon platensis, Stegodon florensis, Stegodon orientalis and Stegodon trigonocephalus.* Data are from PHYLACINE 1.2 [71]. Distributions for species are based on models of where these species would live presently and without anthropogenic pressures, indicating that species richness of extinct elephants indicates where those species would live today, not where they were historically distributed. (Online version in colour.)

Over the past 50 000 years, we have seen the extinction of a suite of megafauna [79], including elephantids that roamed throughout Eurasia (figure 3) [71,73], which may have acted as alternative hosts and bridged the now disconnected ranges of African and Asian elephants [73]. Would these lost elephantids also have been host to the elephant tapeworm? If so, the elephant tapeworm may be an example of a parasite which has seen a reduction in host richness, but an increase in the mean evolutionary distance among its hosts (figure 1*d*). If true, elephantid extinctions may have changed the host landscape such that the elephant tapeworm is now isolated on two distinct and disjunct host populations. Unfortunately, precise data on historical ranges of hosts is unavailable beyond hindcasted distributional models encompassing large amounts of uncertainty, even for species with prolific fossil records [80]. In the case of the elephant tapeworm, the current distributions of elephant species do not overlap and would not be connected if extinct elephantids roamed the world today (figure 3). However, examining the hindcasted distribution of the woolly mammoth (*Mammuthus primigenius*) (see [81]), this species (and potentially the historical distribution of other extinct elephantids) is likely to have bridged the distributions of the African and Asian elephants.

A more prosaic explanation is that the elephant tapeworm story is simply a case of mistaken identity; that elephant tapeworms in Asian and African elephants are morphologically similar, yet genetically distinct species. Expanding the study by Perera *et al*. [76] to explicitly include tapeworms from African elephants would perhaps resolve this. Currently, poor parasite taxonomy challenges our ability to reconstruct historical and contemporary patterns of parasite sharing, with viruses being particularly problematic as they were historically defined by the host in which they were isolated [82]. As the availability of parasite phylogenies become increasingly available (see [83]), we will be able to identify cases in which parasite evolution is driven by host extinction.

## 5. Non-random extinction and the reshaping of host and parasite assemblages

Extinction is a non-random process, with some clades and some areas more extinction prone than others [84–87]. Since the Cenozoic, mammals have faced extinction as a result of anthropogenic pressures, and climatic and environmental change [80,81]. These impacts have affected large-sized species more intensely [88], and their intensity is non-randomly distributed across space [89]. Today larger-bodied host species and host species with narrow geographic ranges or climatic niche tolerances suffer from disproportionately greater extinction risk [90–95]. Because the attributes that predispose some species to a higher risk of extinction than other species are typically conserved on the evolutionary tree of hosts, the process of extinction can result in a large loss of phylogenetic diversity [1,96] and reshape the phylogenetic tree structure of survivors [97]. These same host traits also covary with parasite richness across host species [98], for example, primates and carnivores with larger body sizes and larger geographic ranges also tend to host more parasite species [99,100]. Thus, the process of extinction may jointly reshape extant host phylogenetic structure and within-host parasite diversity, both mediated through host species traits. However, the direction of trait effects can be complicated: although both large geographic extent and larger body size are associated with higher parasite diversity, hosts with large ranges have reduced extinction risk, whereas hosts with large body size have higher extinction risk.

While host trait predictors of parasite richness have been explored for different parasite taxa [98,101], less work has explored how host traits contribute to variation in the richness of specialist versus generalist parasites. Observations that the relationship between host extinction risk and the ratio of specialist to generalist parasites differs [15] suggest that drivers of parasite loss may differ between these classes of parasite, and thus we might also predict drivers of parasite richness would differ similarly. Testing this prediction requires that we have a robust metric of parasite specificity that is insensitive to recent host extinctions. Exploring how contemporary parasite specificity varies with host traits can provide a potential signal of the effect of non-random host extinction. However, it may simply be infeasible to separate the effects of host traits on determining parasite encounter and transmission from the longer term evolutionary consequences of extinction-driven specialization.

Theory may be of some assistance in separating these effects, clarifying implicit assumptions and guiding future predictions. For example, simple mathematical models suggest that large-bodied hosts are more likely to be infected by generalist parasites than small-bodied hosts. This is based on an assumption that large-bodied hosts are a better resource for parasites, thus making the cost of generalism (poorer adaptation to any individual host) easier to pay [102]. This would suggest that biased extinctions of large-bodied hosts may more likely result in increases in apparent specificity, rather than in coextinction. However, this model also identifies cases where that pattern could reverse, and large-bodied hosts would be more likely to be infected by specialist parasites. Empirically, there is evidence for large-bodied hosts being more heavily infected by generalist parasites in some systems [102] and more heavily infected by specialist parasites in other systems [103–105].

## 6. Ghosts of future extinctions

**Figure 4.**
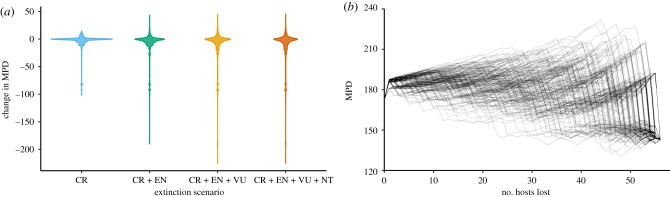
(*a*) Changes in host specificity measured as the mean pairwise phylogenetic distance among hosts (MPD) as hosts are removed according to their IUCN status (CR, critically endangered; EN, endangered; VU, vulnerable; NT, near threatened). Extinction scenarios from left to right remove additional mammal hosts according to their current status. To improve visibility, changes in MPD of zero are removed before plotting. These represent parasites with MPD unchanged by future extinction events. Domesticated species and DD species are not assessed by the IUCN and were assigned a status of LC, thus retaining them in each extinction scenario. The host phylogeny and IUCN statuses are taken from PHYLACINE and paired with the host–parasite association data in Farrell *et al.* [65]. (*b*) Changes in host specificity of *Trypanosoma cruzi* measured as the MPD among hosts as hosts are increasingly lost via extinction. Each line represents a single simulation with a different randomized order of extinction for documented hosts, excluding humans and domesticated species. We use *T. cruzi* to illustrate this because it infects a large number and phylogenetic diversity of host species; 200 simulations are depicted. (Online version in colour.)

Considering that the loss of even a single host may impact the apparent host specificity of parasites in multiple ways (figure 1), it is difficult to outline clear predictions for formal comparative analyses investigating the impact of extinction on present-day host specificity. The shift in the phylogenetic signature of a parasite across the host phylogeny will depend on which host species is lost from the phylogeny, and different parasites will be impacted differently with the loss of the same host species, depending on their initial phylogenetic host range. However, we may study the impacts of extinction on host specificity through the lens of the current biodiversity crisis. Parallel to earlier studies examining the potential for parasites to go extinct with the loss of their hosts [16,50,106], we may similarly erode existing host–parasite networks and examine resulting impacts on host specificity; however, these approaches tend to ignore the potential for parasite host-switches. To demonstrate, we can examine future impacts of biodiversity loss on the host specificity of mammal parasites by removing sets of hosts based on their IUCN status, with all critically endangered hosts removed first, followed by those in categories with the decreasing risk of extinction (figure 4*a*). Exploring the mean pairwise phylogenetic distance among hosts (MPD) as a metric of host specificity, we see that the majority of parasites experience little change with future host extinctions, but there are a few with large changes in MPD. As additional hosts with a lower risk of extinction are lost, more extreme reductions in MPD are seen, while other parasites will see increases in MPD.

In the previous example, all hosts are removed simultaneously, based on their risk of extinction, but in reality, host extinctions will have an ordering, which will result in different trajectories for changes in phylogenetic host specificity as hosts are lost. Figure 4*b* illustrates the variable trajectories that shifts in MPD can take as the hosts for a single parasite go extinct. Each line represents a single randomized order of host extinction, indicating that the order of host extinction may result in increases or decreases in apparent specificity. While this is a simple example to illustrate this phenomenon, future studies may examine these patterns in increasingly realistic contexts of non-random and projected host extinctions, or consider simulated extinctions in the context of a host community network and incorporating additional interactions among hosts.

**Figure 5.**
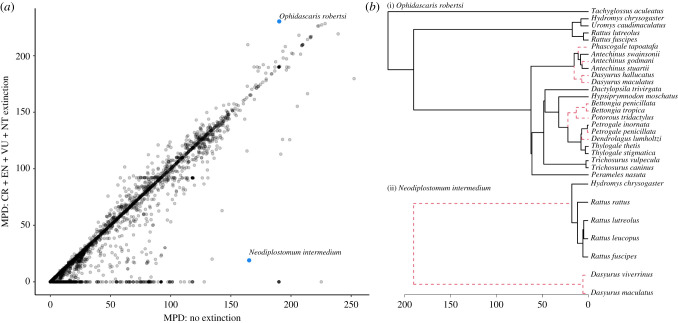
(*a*) Host specificity of parasites, measured as the mean pairwise phylogenetic distance (MPD) among contemporary hosts (*x*-axis), and assuming the extinction of all mammals except those categorized as LC or DD by the IUCN (*y*-axis). (*b*) Examples of future host extinctions on the phylogenetic relationships among hosts for (i) *Ophidascaris robertsi* and (ii) *Neodiplostomum intermedium*. Extinct lineages are denoted by red dashed lines and represent the loss of species assigned IUCN categories other than LC (host phylogeny and IUCN statuses are taken from PHYLACINE and paired with the host–parasite association data in Farrell *et al.* [65]). Scale bar represents millions of years. (Online version in colour.)

To further explore projected changes in host specificity for particular parasites, we examine differences in MPD as a measure of host specificity among extant hosts and after projected host extinction (figure 5*a*). Assuming a simulated extinction event leaving only hosts assessed as least concern (LC) or data deficient (DD) by the IUCN, we see that the majority of parasites fall on the 1 : 1 line, indicating that future extinctions will not have a consistent directional impact on phylogenetic host specificity. Nonetheless, phylogenetic specificity will change for a large number of parasites. Among those parasites impacted, some generalists will be reduced to single-host parasites (those with MPD of zero after host extinction), some will become ‘apparent specialists' (reduction in MPD) and others will become ‘apparent generalists’ (gains in MPD). Examples of increasing apparent specialism and generalism can be seen with extinctions among the hosts of the nematode *Ophidascaris robertsi* and the trematode *Neodiplostomum intermedium* (figure 5*b*). Both parasites infect Australian mammals including marsupials and native placental rats. However, future host extinctions are likely to trim away internal branches among hosts of *O. robertsi* leading to increased phylogenetic distances, while all of the marsupial hosts of *N. intermedium* will be lost and lead to greatly increased phylogenetic specificity. Although the number of projected host extinctions is high, the ecology of *O. robertsi* may be relatively unimpacted as extinctions do not prune large swathes of the host tree, multiple sister taxa are projected to survive, and mammals are only intermediate hosts for this parasite which uses pythons as a definitive host [107]. However, as *N. intermedium* uses mammals as definitive hosts, the large phylogenetic distances between Australian eutherian rats and marsupial hosts could mean that the projected extinction of the *Dasyurus* hosts will dramatically shift the selective landscape of the parasite.

## 7. Impacts of host extinction on parasite ecology and evolution

Host extinction and the coextinction of dependent parasites will impact the structure and function of ecosystems [3,19,108] and may shift the distributions of zoonotic diseases [18]. In addition to complete host loss, there can be large impacts due to changes in host populations as they decline to extinction. These include reductions in host and parasite abundance, leading to reduced population densities or range sizes. At the extreme, for parasites that are ‘obligate’ multi-host parasites (where ‘obligate’ refers to a situation where the net reproductive rate of the parasite on any individual host is less than one, so that parasite maintenance requires multiple hosts; [23]), host population declines may lead to parasite extinction well before any host actually goes extinct. We are already seeing evidence of such changes in many host populations [2,109,110], and these host declines have been marked by the loss of parasites in threatened species [14,111] and changes in the proportion of generalist versus specialist parasites in some host groups [15]. In the latter case, these changes likely result from shifting intra- and interspecific contact rates among hosts, which may have proximate impacts such as shifting parasite distributions, population sizes and relative rates of host exposure. While it is clear that host extinction will influence parasite abundance, whether extinction increases or decreases transmission will depend on specifics of the system and how it impacts the relative abundance of competent hosts. In instances where parasites lose hosts that support onward transmission, we may see reduced transmission potential, whereas the extinction of off-target or dead-end hosts may allow for the maintenance of robust parasite populations within more competent reservoir hosts. Further, parasite life histories, such as transmission mode, may evolve in tandem with shifting host specificity [112], and are likely to mediate this effect. For many parasites, transmission is only weakly or not impacted by reductions in host density, and in extreme cases, such as vector-borne or strongly frequency-dependent transmission, reduced host density can improve transmission [113,114].

### (a) Transmission frequency

Whether host extinction increases or decreases parasite transmission will impact changes on evolutionary timescales [115] and may impose new selection pressures on parasite evolution [116]. For example, host extinction may limit gene flow among previously connected parasite populations, promoting specialization of parasites on their newly isolated hosts. For many infectious organisms, and especially those with short generation times such as viruses and bacteria, this isolation could lead to allopatric speciation, a process that would be reflected in congruent tree shapes in co-phylogenetic analyses [83,117]. This process of host extinction leading to parasite specialization and speciation may be quite common, but the lack of robust parasite fossil records and data on historical hosts make this difficult to identify. Future co-phylogenetic methods may benefit by modelling the impacts of host extinctions, as reconstructions may be differentially impacted by the loss of closely versus distantly related host species [118]. For relatively long-lived parasites, such as cestodes, including the elephant tapeworm discussed above, we may be able to identify examples where parasites are in the process of speciation. The longevity of adult tapeworms in their definitive hosts is quite variable, surviving from weeks to multiple decades up to the lifespan of the host [119]. The long generation times of some tapeworms might not allow sufficient time for divergence following historical extinctions and subsequent geographic isolation of their host species. This may be the case for the elephant tapeworm, but further research on maximum longevity, population genetics, and phylogenetic analyses of both the Asian and African populations would be needed.

### (b) Costs of generalism

As host extinction drives increasing phylogenetic isolation of host species, this is likely to alter the costs of generalism, potentially promoting further parasite specialization and speciation, and also shift the optima for virulence and transmission across extant hosts [33,62,120]. Multi-host parasites are often assumed to experience a cost of generalism, the increased transmission opportunities associated with additional host species trading off against fitness benefits gained by specializing on any particular host species [62,120–122]. Costs of generalism can take two forms; one is a more global cost in which having multiple hosts reduces the potential for coevolution with any one host, meaning generalists may not be as well adapted to their hosts, on average, when compared to specialist parasites. The other form that a cost of generalism may take is greater variation in fitness across hosts, with parasite adaptation to novel hosts resulting in reduced fitness in original hosts [123], with the magnitude of this trade-off increasing with the phylogenetic distance between hosts [62]. Due to either or both of these costs, generalist parasites are therefore likely to have lower fitness in any given host than is possible in a single-host relationship, which is offset by the demographic advantage of an expanded reservoir of available hosts [124]. In this context, the influence of host extinction on parasite mean fitness will depend precisely on which hosts are lost, the evolutionary distances between extant hosts and the types of costs of generalism that were being paid (e.g. if they were reasonably well adapted to any host in the system).

### (c) Virulence

Parasite fitness relies on successful transmission, which requires the exploitation of host resources and ultimately results in damage to hosts, termed ‘virulence’. For many parasites, greater host exploitation facilitates increased transmission, but if viruence is too high, then the transmission may be reduced due to shorter infection duration [125,126]. For multi-host parasites, there may be a unique optimal virulence that maximizes transmission on each individual host [124]. If parasites are constrained to a single level of virulence (i.e. they cannot plastically adjust their strategy to the current host), then parasites will evolve an intermediate virulence, influenced by the relative contribution of each host species to the total force of infection, that maximizes fitness across their host species, but achieves optimal virulence in none [24]. By changing the epidemiological contribution of each species, host extinction is likely to shift the selective landscape for parasites, leading to changes in virulence as parasites adapt to track the optimal virulence of the surviving hosts.

Depending on the relative contributions of different host species to transmission, as well as the optimal virulence within each, the extinction of a particular species may lead to the evolution of increased or decreased virulence on remaining hosts. In table 2, we explore possible evolutionary outcomes of host extinction assuming three host species, the potential for onward transmission in each host, and a single optimal virulence expressed in each host species that maximizes total transmission. Few empirical studies have examined how phylogenetic distance among hosts is linked to parasite virulence, but studies of zoonoses and multi-host domesticated animal parasites found that increased evolutionary distance among hosts is associated with greater potential for virulence, but at the cost of reduced transmission [33,34]. Predicting the evolution of virulence in multi-host systems is a complex challenge, but as biodiversity loss dramatically restructures host–parasite associations, and humans become increasingly isolated in the tree of life, understanding how parasite virulence may evolve in response to host extinction is increasingly important.

**Table 2.** Examples of how parasite virulence might evolve in response to host extinction. The first column indicates the initial state of each system prior to extinction, including the optimal virulence for each host clade if this was the sole host, and the evolved optimal virulence expressed across all hosts. In these examples, optimal virulence is skewed towards the single-species optimum for the host clade that contributes the most to the force of infection. The second and third columns outline the shifts in the system resulting from two extinction scenarios in which the phylogenetic distances among hosts is either decreased or increased. With the extinction of a given host, in general, we would expect virulence to evolve towards the optimal virulence for the remaining species, though this is dependent on the initial state of the system. This framework closely follows the theory in Williams [24].

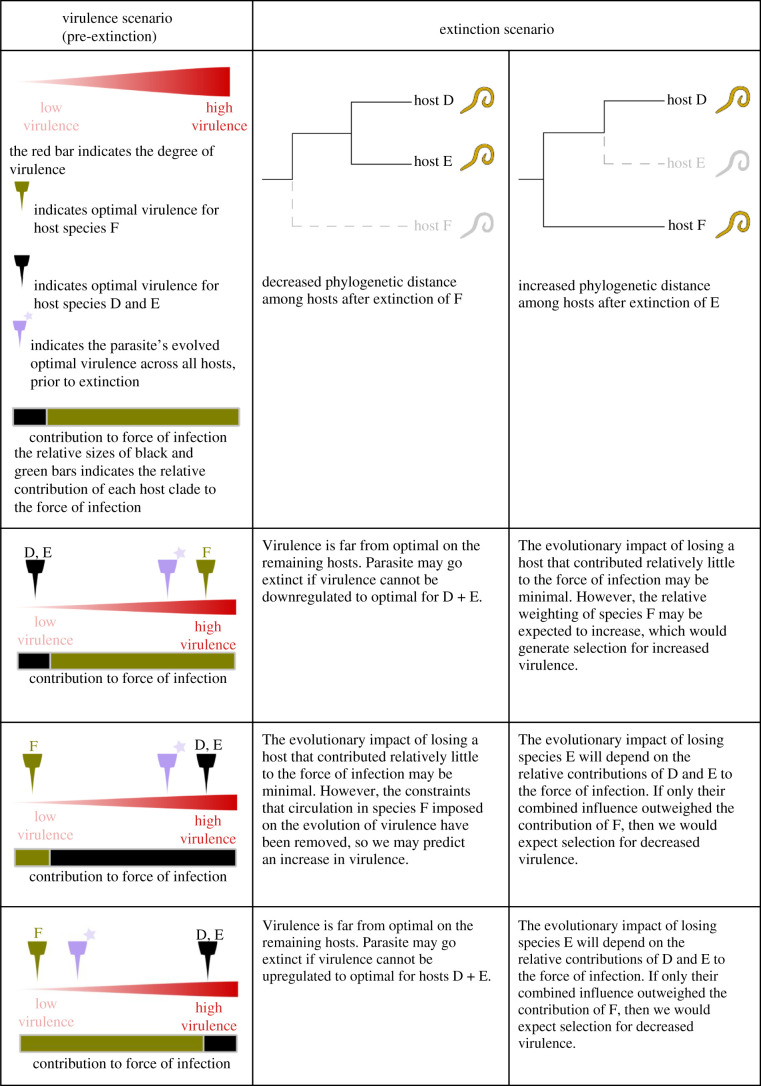

## 8. Conclusion

The current biodiversity crisis is reshaping the tree of life, shifting realized parasite host specificities and the adaptive landscapes of contemporary parasites. Here, we demonstrate that the impacts of host extinction on phylogenetic measures of host specificity are context-specific, with host extinction potentially leading to both increases and decreases in generalism of parasites. We suggest that these changes in specificity are likely to have complex impacts on parasite evolution, including further evolution of specialist or generalist strategies, and the shifts in parasite virulence. We show that past extinctions may have reshaped host–parasite associations, and thus care should be taken when drawing inference from present-day patterns of host specificity. In the case of more recent host extinctions, parasites today may appear more or less specialized, masking an intrinsic ability to infect novel host species, and altering our perceptions of their potential host ranges.

Just as past extinctions have shaped present-day host–parasite interactions, ongoing biodiversity loss will continue to shape disease dynamics into the future. Beyond extinction, climate change-induced range shifts may promote host–parasite sharing and novel interactions never seen before in evolutionary history [127]. Infectious diseases act as synergistic drivers of host extinction, with impacts due to infectious diseases increasing as populations decline to extinction [128]. Host extinction is likely to decrease global parasite richness through the coextinction of specialist parasites [3], but generalist parasites are most often associated with host declines [129]. The relative loss of specialist parasites may remove protective effects of co-adapted parasites and expose hosts to more virulent parasites through the reduction of immune cross-protection and opening of new niches for generalist parasites [130]. When shifting to novel hosts, parasites may display increased virulence due to a lack of coevolutionary history between host and parasite [131], and host extinctions may also select for increased parasite virulence in some systems, exacerbating disease-mediated host declines. While the current theory is well developed for single-host single-parasite systems, expanding on theories of host specificity, co-adaptation, and virulence evolution in multi-host systems is crucial for better understanding how biodiversity loss impacts infectious diseases, and mitigating disease impacts as we navigate the current biodiversity crisis. We note that many of the concepts discussed here for host–parasite systems may also be applied to symbionts in general, offering new avenues for future research into the cascading impacts of host extinction.

## Data accessibility

Data and R scripts to reproduce the figures are available at https://github.com/DiseaseMacroecology/ghost-host and doi:10.6084/m9.figshare.14573787.
